# The International Congress on Tuberculosis

**Published:** 1908-08

**Authors:** 


BUFFALO MEDICAL JOURNAL.

A Monthly Review of Medicine and Surgery.

*EDITOR:*

WILLIAM WARREN POTTER, M. D.

All communications, whether of a literary or business nature, books for review and exchanges, should be addressed to the editor 238 DELAWARE AVE., BUFFALO, N. Y

VOL. LXIV. AUGUST, 1908. No. 1

### The International Congress on Tuberculosis.

THE most important meeting in the year insofar as it bears on the question of preventive medicine is the one given in the title of this article. It will be held in Washington beginning September 21, and continuing until October 12, 1908, thus covering a period of three weeks. We deem the congress of such great importance that we devote considerable editorial space to this notice, the material for which is furnished by the secretary-general, Dr. John S. Fulton, whose headquarters are in the Colorado Building at Washington.

One of its most important features is the fact that the President of the United States has accepted the presidency of the Congress. The President's letter to Dr. Lawrence F. Flick is so comprehensive, and withal so clearly conclusive as to the great work devolving upon this assemblage of scientific men, that we publish its full text.

THE WHITE HOUSE,
*Washington, May* 12, 1908.

SIR: It is with great pleasure that I accept the presidency of the International Congress on Tuberculosis which is to meet in this city on September 21, 1908, and extend its session to October 12, 1908. Official duties, however, may prevent my presiding at the initial meeting of the congress, in which case I will deputise secretary Cortelyou.

The importance of the crusade against tuberculosis, in the interest of which this congress convenes, cannot be overestimated when it is realised that tuberculosis costs our country two hundred thousand lives a year, and the entire world a million lives a year, besides constituting a most serious handicap to material progress, prosperity and happiness, and being an enormous expense to society, most often in those walks of life where the burden is least bearable.

Science has demonstrated that this disease can be stamped out, but the rapidity and completeness with which this can be accomplished depend upon the promptness with which the new doctrines about tuberculosis can be inculcated into the minds of the people and engrafted upon our customs, habits and laws. The presence in our midst of representatives of world-wide workers in this magnificent cause gives an unusual opportunity for accelerating the educational part of the program.

The modern crusade against tuberculosis brings hope and bright prospects of recovery to hundreds and thousands of victims of the disease, who under old teachings were abandoned to despair. The work of this congress will bring the results of the latest studies and investigations before the profession at large and place in the hands of our physicians all the newest and most approved methods of treating the disease—a knowledge which will add many years of valuable life to our people and will thereby increase our public wealth and happiness.

The International Congress on Tuberculosis is in the interest of universal peace. By joining in such a warfare against a common foe the peoples of the world are brought closer together and made to better realise the brotherhood of man; for a united interest against a common foe fosters universal friendship. Our country, which is honored this year as the host of other nations in this great gathering of leaders and experts and as the custodian of the magnificent exhibit which will be set up by the entire world, should manifest its appreciation by giving the congress a setting worthy of the cause, of our guests, and of ourselves. We should endeavor to make it the greatest and the most fruitful congress which has yet been held, and I assure you of my interest and services to that end.

With expressions of appreciation for the compliment conferred in extending the invitation to become president of the congress.

Very respectfully,

THEODORE ROOSEVELT.

The congress meets once in three years and after 1908 will not meet in this country for many years to come. It, therefore, furnishes an opportunity for Americans to show what the western world can do in the way of a host, as well as what they can contribute to the scientific prevention of an infectious disease that has invaded every corner of the earth. Moreover, it will be a real world's congress in which delegates will discuss the tuberculosis problem for three weeks, the participants being some of the most eminent authorities on the subject.

The congress will be divided into seven sections, thus giving ample scope for the consideration of every phase of the problem, in which both scientific and lay members may participate. A tuberculosis exposition will furnish entertainment and instruction; clinics and demonstrations will give object lessons to the delegates; and valuable publications will be issued, chiefest of which will be the transactions of four volumes, free to all members who pay the fee of five dollars. Associate members may pay a fee of two dollars, thus acquiring all the privileges of membership except the right to vote and to receive a copy of the transactions.

All persons who desire to become members should address Dr. John S. Fulton, secretary-general, 714 Colorado Building, Washington, D. C.

---

CALOX, the oxygen tooth powder, is commanding the attention of the general public. as well as of the profession. The chemistry of the dentifrice has the approval of experts, its composition is as near perfect as the chemical laboratory can make it, and altogether it is the most hygienic tooth powder with which we are familiar. Moreover, nothing can approach it as a cleanser of artificial dentures. The oxygen it contains does its work.

---

THE American navy has been given its woman nurse corps by act of the recent congress, but now Great Britain goes us one better by proposing for its army a mounted nurse corps. There is now in existence a company of young women which has been trained by a veteran officer and will be ready for the test. The Islington Drill Brigade Girls' yoemanry they are called, and now are twenty-five strong and efficient riders. The girls wear the ordinary military rank badges, surmounted by a spur and cross whips; one tunic blouse, with a blue skirt with white braiding round the bottom; white gauntlets, black leggins and a yellow sash. A red and blue field service cap is worn with a chin strap. Riding-whips are carried.

---

THE National Volunteer Emergency Service, instituted in 1900, has recently been reorganised by the election of Dr. James Evelyn Pilcher, the distinguished editor of *The Military Surgeon,* as its Director General, and Dr. F. Elbert Davis, of New York, as its Adjutant General. Its work will be conducted along military lines, the details being worked out in three separate Corps, a First Aid Corps, a Public Health Corps, and a Medical Corps—the latter consisting of physicians, with rank from Lieutenant to Colonel, according to length of service, to whom are afforded special opportunities for emergency training. It includes among its personnel a large number of notable personages, and is rapidly extending its membership throughout the country. Full details regarding the service and its great work may be obtained by addressing Director General Pilcher at Carlisle, Pa.

The health authorities of Chicago, in common with those in many other cities, are waging a vigorous war on the house fly. Dr. W. A. Evans, health commissioner, thus characterises the insect: "He is the filthiest, most dangerous and most common of disease-spreading insects that infest these parts in summer. He is born in and lives on decaying vegetable and animal matter. He was a maggot before he was a fly. It is only natural that he should carry thousands of disease germs on his feet and deposit them in the food you eat. Doubtless much sickness and many deaths can be prevented by keeping the fly out."

---

Paper milk cans are coming into use all over England. They are never used twice—hence hygienic. They are lighter—hence liked by those who handle them. They reduce the noise of the matutinal milk cart—hence weary victims of insomnia bless them.

---

The University of Buffalo, through Vice-Chancellor Charles P. Norton, has closed a contract with the County of Erie for the transfer of land which has been the subject of negotiation for some months past. The plot includes about 104 acres known as the almshouse property and $5,000 has already been paid by Vice-Chancellor Norton, as a part of the purchase money.

